# Correction: Experimental and theoretical investigation of a mesoporous NiCo_2_O_4_/rGO nanosheet network as a high-performance anode for lithium-ion batteries

**DOI:** 10.1039/d5ra90087e

**Published:** 2025-07-15

**Authors:** Muhammad Inayat Ullah, Tanveer H. Bokhari, Rimsha Aqeel, Saqib Javed, Zahid Abbas, Shakeel Abbas, Atia Khalid, Athar Javed, Shafqat Karim, Rao Tahir Ali Khan, Amjad Nisar, Mashkoor Ahmad

**Affiliations:** a Nanomaterials Research Group, Physics Division PINSTECH Islamabad 44000 Pakistan mashkoorahmad2003@yahoo.com chempk@gmail.com; b Department of Chemistry, Government College University Faisalabad Pakistan; c Theoretical Physics Division, PINSTECH Islamabad 44000 Pakistan; d School of Materials Science and Engineering, Tsinghua University Beijing China; e Department of Physics, University of the Punjab Lahore, 05422 Pakistan

## Abstract

Correction for ‘Experimental and theoretical investigation of a mesoporous NiCo_2_O_4_/rGO nanosheet network as a high-performance anode for lithium-ion batteries’ by Muhammad Inayat Ullah *et al.*, *RSC Adv.*, 2025, **15**, 20321–20329, https://doi.org/10.1039/D5RA01695A.

The authors regret that the affiliation for Tanveer H. Bokhari was incorrectly shown in the original manuscript. The authors would like to clarify that affiliation *b* is the only affiliation for Tanveer H. Bokhari. The corrected list of affiliations is as shown here.

The Royal Society of Chemistry apologises for these errors and any consequent inconvenience to authors and readers.

